# Implementation of the evidence for the improvement of nursing care to the critical patient’s family: a Participatory Action Research

**DOI:** 10.1186/s12913-018-3177-8

**Published:** 2018-05-11

**Authors:** Laura de-la-Cueva-Ariza, Pilar Delgado-Hito, Gemma Martínez-Estalella, Gemma Via-Clavero, Teresa Lluch-Canut, Marta Romero-García

**Affiliations:** 1 Fundamental and Medical-Surgical Nursing Department, Nursing School (Faculty of Medicine and Health Sciences), University of Barcelona, Barcelona, Spain; 2 Hospital Clínic, Barcelona, Spain; 3 Intensive Care Unit. Hospital Universitario de Bellvitge, Barcelona, Spain; 4 Public Health, Mental Health and MCH Department, Nursing School (Faculty of Medicine and Health Sciences), University of Barcelona, Barcelona, Spain

**Keywords:** Nursing, Intensive care, Family, Knowledge, Patterns of knowing, Qualitative research, Participatory action research

## Abstract

**Background:**

There are many descriptive studies regarding the needs of the family, as well as those regarding nursing care aimed directly at family members. However, there is no widespread application of such evidence in clinical practice. There has also been no analysis made of the evolution of patterns of knowing during the act of improving clinical practice. Therefore, the purpose of the study is to understand the change process aimed at improving care to critical patient’s families, and to explore the evolution of patterns of knowing that nurses use in this process.

**Methods:**

Qualitative study with a Participatory Action Research method, in accordance with the Kemmis and McTaggart model. In this model, nurses can observe their practice, reflect upon it and compare it with scientific evidence, as well as define, deploy and evaluate improvement strategies adapted to the context. Simultaneously, the process of empowerment derived from the Participatory Action Research allows for the identification of patterns of knowing and their development over time. The research will take place in the Intensive Care Units of a tertiary hospital. The participants will be nurses who are part of the regular workforce of these units, with more than five years of experience in critical patients, and who are motivated to consider and critique their practice. Data collection will take place through participant observation, multi-level discussion group meetings and documentary analysis. A content analysis will be carried out, following a process of codification and categorisation, with the help of Nvivo10. The approval date and the beginning of the funding were December 2012 and 2013, respectively.

**Discussion:**

The definition, introduction and evaluation of care strategies for family members will allow for their real and immediate implementation in practice. The study of the patterns of knowing in the Participatory Action Research will be part of the theoretical and practical feedback process of a professional discipline. Also, the identification of the construction and evolution of knowledge will provide decision elements to managers and academics when choosing strategies for increased quality.

## Background

Intensive Care Units (ICU) in different countries, particularly in southern Europe, are often closed spaces that are regulated by health professionals, where internal organisation is designed without the participation of patients and their families [1, 2]. The usual pattern of these units is restrictive with rigid informative guidelines regarding visiting hours [3–7] and internal dynamics, thus promoting a significant separation, with family members on the one hand and healthcare teams and the patient on the other [2, 8, 9].

In this context, technology and the complexity of the critical patient influence the development of technical skills and nurse care methods in the ICU. Sometimes, this involves the prioritisation of care focused on physical and technical aspects centred around the patient as an object of care, without taking into account that he or she is part of a family unit [9–19].

All members of this family unit are deeply interconnected, and thus any unfavourable situation affecting one member has a negative effect on the others, destabilising the family and causing a crisis situation [17, 20, 21]. In this sense, the hospitalisation of a person in the ICU provokes an alteration of family structure, causing a series of disruptions and feelings, which have been widely discussed in literature, comprising of an experience that is almost always described as traumatic. When this happens, the members of the family must confront emotional, cognitive, and social stressors that generate feelings of shock, uncertainty, denial, anger, despair, hope, guilt, anxiety and fear of a family member’s death [9, 14, 15, 17, 20, 22, 23]. Therefore, these stressors appear in a context in which the family sees their coping abilities exceeded, as its members also become recipients of nursing care with a series of requirements that need to be met.

The needs of relatives appear not only at the time of admission to the ICU, but rather during the entire length of hospitalisation until the patient is no longer considered critical [22, 24]. The identification of these needs has a large consensus among the various authors who have studied the topic [9, 14, 16, 17, 19, 20, 22–30]. The following list is a synthesised summary of some of the most noteworthy needs: (1) Receive honest and understandable information with respect to the clinical condition of the patient. (2) Have hope, whether with regard to a recovery or a dignified and painless death, and to have time to spend with the family member. (3) Have proximity with the patient. (4) Be relieved of anxiety. (5) Feel that the patient is well cared for and that the skilled and competent unit professionals genuinely care for the patient. (6) Be able to reassure, support and protect their loved one. (7) Have some degree of comfort during the process.

On the other hand, the family has resources that can promote the welfare of the critically ill person, provide support in managing situations and provide resources to members of the health team, thus, increasing the possibilities of intervention on the patient [11, 31, 32]. There are even studies that point to a better prognosis of patients when the family is involved in the care and when the nurse has an active role in supporting families [33]. Ultimately, attending to the family means better care to the patient [34].

Despite the evidence above, studies confirm that family members and nurses have different perceptions of the situation [2, 35]. Various investigations concur that nurses have ambivalent feelings and beliefs with regard to the family of the critical patient [1, 2, 14, 22, 34–37]. In this sense, nurses recognise that the family has to be attended to offer comprehensive care to the patient, but, at the same time, they consider them an external element to the unit, a source of stress (leading to limited contact) and a difficult workload to take on.

On the basis of this ambivalence, in recent years there have been various changes made in order to provide families of critical patients with appropriate care [32, 38–40]. Despite this, in the context of Spain, interventions are scarce and relate to the arrival protocol of the family during admission [2, 10, 41], open visiting hours [36], recommendations for the involvement of the family in the care of the critical patient [11], and the improvement of involvement with the family in a consensual and general manner [2, 19, 24, 42].

In this context, the transition towards implementing and evaluating care in a systematic way has not been carried out, despite the large amount of replicative and descriptive studies that outline the situation and the needs of the family, as well as studies on nursing care in other countries [16, 43]. Therefore, there is a real need for families to be included in the care process; defining, implementing and evaluating the most complete care needs, which are truly holistic and developed for the families of the critical patient, just as this study is claiming.

It is not easy to change clinical practices in healthcare environments that are as complex as an ICU, even when implementing recommendations that are derived from evidence, and this is a problem that has been recognised internationally [2, 44–46]. It is difficult to urge nurses to adopt changes that do not reflect their daily reality, requiring them to fully engage with investigations in which they have a passive role [10]. Consequently, in order to achieve real progress and change, this study forms part of a Participatory Action Research (PAR) that values knowledge, experience and opinions of nurses, empowering them [47] to change their practices.

Thus, through the process of PAR, nurses not only improve the care they offer but they implement and develop knowledge [18]. Knowledge is not something static; it is dynamic and changeable, and patterns of knowing within a discipline reflect its progress and maturity [48]. In order to understand the nature of nursing knowing, it is important to bear in mind that nursing is a professional discipline whereby nurses obtain part of their disciplinary knowledge via formal and informal education, and expand this acquired knowledge via clinical experiences, thus creating various dimensions of knowing [49–52].

Nurses use various sources of knowing in their everyday practices. The nature of which has been explored by several authors who conclude that, in addition to scientific knowledge, nurses use other patterns, or models, of knowing, which are essential to their practice [51–54]. Initially, Carper [53] identified four basic patterns of knowing: (1) Empirical (from a research process that describes, explains and predicts the phenomena of interest in the discipline of nursing); (2) Aesthetic or the Art of Nursing (makes possible knowing what to do and how to be in the moment, instantly, without conscious deliberation); (3) Ethical (encompasses the moral component of nursing) and (4) Personal (relative to knowledge of the Self and others in relationship).

Subsequently, White [54] added the pattern of socio-political knowing, and Chinn & Kramer [51, 55] studied and reformulated the fundamental patterns, elaborating their “Integrated Theory” in which a new pattern, the emancipatory, emerges from praxis and through the development of the other four patterns.

Research pertaining to the production of nursing knowledge and, in particular, patterns of knowing, has been basically implemented in the Anglo-Saxon context. Qualitative methodologies have been used via the analysis of narratives [56, 57] and quantitative methodologies via the use of “Nursing Patterns of Knowing Scale”, for example, developed by Rubarth, Reed and Guadron [58, 59]. The Spanish context has only studied the production of nursing knowledge from practice and via reflectivity [18]. But no studies have been found that analyse the evolution of patterns of knowing in the context of a participatory and reflexive methodology, which involve the implementation of nursing knowledge.

According to Chinn and Kramer *“The full range of possible patterns of knowing and approaches to knowledge development remain to be developed, and those currently named require refinement [...] which is imperative for development of disciplinary nursing knowledge”* [51]. This quote is relevant as patterns of knowing have not been explored in a process that naturally involves praxis (action-reflection) and decision making in order to change and improve practices. Studying the process of mobilisation and production of knowledge can increase the general body of disciplinary knowledge through feedback between the practice and theory of a professional discipline such as nursing.

## Methods

### Aims

1. To understand the process of change aimed at improving the care provided to relatives of the patient in a critical situation, in the intensive care unit of a tertiary hospital.
2. To explore the evolution of patterns of knowing that nurses use in a process of action-research for the improvement of nursing practice, in relation to the family care of the critical patient in the ICU of a tertiary hospital.

#### Objectives

- 1.1 Analyse the characteristics of family care in the polyvalent ICU of the tertiary hospital.
- 1.2 Identify elements of change and improvement of nursing care provided to the family of the critical patient, derived from the reflection process.
- 1.3 Assess the effects of new practice on family and nurses from the point of view of the latter.
- 2.1 Identify the patterns of knowing used by the nurses for decision-making with regard to improvements of care to family members.
- 2.2 Analyse the evolution of the different patterns of knowing during the process of Participatory Action Research.

## Design

The paradigmatic perspective that guides this study is constructivist. This paradigm recognises the collective construction of knowledge [60]. This knowledge-construction is produced by a gradual consensus that allows us to compare, contrast and negotiate each individual element dialectically [61]. Thus, a creative process is developed whereby participants establish their day-to-day life and negotiate, through a process of (co) construction, what they think is the best way to live [62], ultimately creating legitimate knowledge reconstructed by those who are going to use it.

A qualitative methodology with a method of Participatory Action Research (PAR), according to the model proposed by Kemmis and McTaggart [63], was chosen to achieve the objectives of this study. This model is made up of four stages: planning, action, observation and reflection. These stages are grouped into cycles, which can be repeated in a spiral shape.

### Setting of the study

The study will be carried out in a tertiary hospital in Barcelona (Spain). The specific context will be 3 multi-purpose ICU, with a nurse-patient ratio of 1:2. The standard organisation of these units has a visitation schedule of three periods a day; relatives entering the unit are restricted to two people per patient, and information is given only once per day, the physician being responsible for providing this update. Sometimes these measures are made flexible under special circumstances: deterioration of the patient, situation of imminent death, etc.

### Participants

Potential participants in the study will be nurses who work in four shifts: one in the morning, one in the afternoon and two at night. Theoretical sampling will be carried out to attract participants with the following characteristics: (1) Nurses who form part of the ICU’s standard workforce. (2) Nurses with a minimum of 5 years of experience in caring for critical patients. (3) Nurses who are motivated to reflect upon and critique their practice. This way, participants with assured participation in all stages of the study, who recognise the current situation with reference to the issues that will be worked on, and who have the ability to establish improvements with regard to attention to family members of critical patients will be recruited.

To recruit the different participants, a meeting with the Hospital’s head of Training, Teaching and Research will be held, so that he or she can assist in the selection of nurses who meet the predefined criteria. Subsequently, meetings will be scheduled with the pre-selected nurses to explain the purpose and methodology of this project and confirm their participation.

With the objective that change is implemented and experienced by the greatest number of nurses possible, discussion groups with a multi-level design will take place [64]. Two levels of participation will be established: members of the support group and members of the core group [19].

With an aim to ensure discursive productivity and achieve further consensus [64, 65], participants will be distributed in different groups on the basis of the criteria of homogeneity (nurses who form part of the ICU regular workforce, with the same recurring shift pattern and who are motivated to reflect upon and critique their practice) and heterogeneity (nurses who develop their work in different UCI with several years of experience in the critical patient care, with varying degrees of formal training).

Firstly, four support groups will be formed under the criterion of working shifts, in order to facilitate meetings between the members of each group. It is estimated that the number of members in each support group will be a minimum of three and a maximum of nine [64], so as to ensure the fluidity of communication between them.

**Fig. 1:**
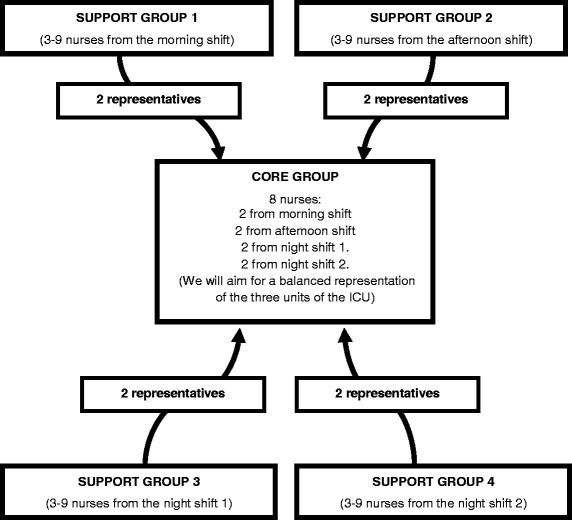
Methods of participation in the study (multilevel discussion groups)

Subsequently, the participants in each support group will elect two representatives to form part of the core group, which will be comprised of eight nurses, as recommended by Morgan [66]. Members of this group will be responsible for transmitting the reflections and consensus of ideas and proposals arising from meetings of the core group to members of the support group that they represent, and vice versa (Fig. 1).

#### Sample size estimation

The number of participants in the discussion groups (support groups) defined in the literature [64] and the representativeness in each group of the criteria of homogeneity and heterogeneity described above, were taken into account to estimate the sample size. Therefore, in order to ensure a productive discourse and consensus among the participants, a sample size between 12 and 36 nurses is estimated.

### Development of the PAR model’s phases

The study will consist of a preliminary phase, called reconnaissance, and of two cycles within a spiral, with its four corresponding stages, as described by Kemmis and McTaggart [63] in their PAR model.

#### Reconnaissance phase

In this phase the researchers will carry out the following actions: (1) Observation of nursing practice in relation to the intervention aimed at the family, through a participant observation with a type III role (observer-as-participant) according to the Junker classification adapted by Valles [65]. (2) An analysis of nursing records pertaining to nursing interventions with the families of critical patients.

#### First cycle

##### Planning stage

Two meetings will be held, each with a maximum duration of 2 h, with the following objectives: First, agree on the definition of the roles of participants and researchers [10, 67, 68]. Plan how the participants will carry out the self-observation and individual reflection of their practice. And, finally, provide documentation on the methodology of the study to participants.

##### Stages of action and observation

During these stages, nurses will carry out self-observation of their practice in a minimum of three situations of interaction with family members. Subsequently, their observations will be recorded in the field diary via a narrative of the observed situation [57, 69] and on the basis of the three dimensions defined by Kemmis and McTaggart [63]: language, activities and relationships established with the family. And, finally, reflections arising from the confrontation of the reality of their interventions will be recorded alongside the scientific evidence relating to the issue and the identification of the various patterns of knowing.

##### Stage of collective reflection

Based on the reflections written by each nurse, a collective reflection process will begin, consisting of two phases: support group meetings and core group meetings. During these meetings, the emerging issues deriving from self-observation, individual reflections and collective reflection resulting from the reanalysis of the significance of the current situation will all be pooled together, to become the basis of a revised plan that will allow to start the second cycle of action-reflection.

#### Second cycle

##### Re-planning stage

At this stage, support groups will meet to develop viable and realistic change strategies, taking into account their daily realities. These strategies will be transmitted and agreed upon with other participating members of the core group.

##### Stages of action and observation

This consists of implementation in practice, self-observation and reflection on change strategies and patterns of knowing put into action.

##### Stage of collective reflection

Using the same phases of this stage of the first cycle, participants will analyse what has happened, what is expected, the limitations and the consequences in relation to the strategies for change, and patterns of knowing used and developed during the process.

### Data collection

Following the principles of qualitative design, data and its analyses will be collected simultaneously [70]. The techniques for obtaining information to be used are: participant observation, multi-level discussion groups, documentary analysis and an ad hoc questionnaire for the description of the population and the sample.

#### Participant observation

The principal investigator will perform a participant observation of the nursing practice following the recommendations of Spradley [71]. This technique will be comprised of observing situations of nurse-family interaction, with the objective of forming a diagnosis of the nursing intervention with the family. This will be carried out during family visiting hours, in the three different shifts, within the hospital’s different polyvalent UCI units, until reaching the theoretical saturation of data. It is estimated that this observation period will go on for approximately one month. The observer will use an observation grid in order to facilitate the registration of language (verbal and non-verbal), activities and relationships of each nurse with the family.

One of the strategies that will be used to minimise observer bias will be to make observations prior to recruiting participants for the PAR. The informed consent of the head of service will also be requested, and the health care team will be informed that observations will be carried out regarding general nursing practices, without expressly mentioning the nurse-family interaction. And, finally, observation will be expanded beyond family visiting hours, thus not drawing attention to the observation of solely these family interactions.

The descriptions will be reviewed by a member of the research team, an expert in qualitative research, who will be absent during the observation, to question and to verify whether the description is detailed and of quality.

#### Multi-level discussion groups

All meetings will be recorded (using a digital audio recorder) so that they can then be accurately transcribed and subject to a subsequent analysis. These meetings will be moderated and observed by two members of the research team. The meetings will be held in a room away from healthcare work taking place in the hospital and with less background noise to ensure privacy and a lack of interruptions. This room will allow for the participants, moderator and observer to sit in a circle or oval shape, with an aim to promote intra-group communication [64].

#### Documentary analysis

There will be an analysis of the following documents:

- Nursing records integrated into the chart of each patient, since the beginning of the observation up until data saturation. Only fragments of the records relating to the family will be analysed, excluding any information relating to the patient or nurse.
- The researchers’ field diaries, with respect to the entire research process.
- Participants’ field diaries and narratives.

#### Ad hoc questionnaire for collecting quantitative variables

Socio-demographic variables will be collected (age, gender, years of experience as a nurse, years of experience in ICU, shift work and academic training) in order to describe the population and the sample of participants.

### Data analysis

A thematic content analysis will be carried out, consistent with the constructivist paradigm that guides this research [72]. A process of identification, codification and categorisation of the main axes of underlying meaning in the data will also be carried out. In this sense, via this process of analysis, in addition to describing the obvious and visible components, an abstraction will be created and interpreted, thus accessing the hidden connotations of the data [73]. Nvivo10 software will be used to facilitate this analysis.

### Rigour

According to the trustworthiness criteria defined by Lincoln and Guba [74], the rigour of the research will be guaranteed through: (1) The triangulation of techniques, sources, and researchers. (2) The revision, by participants, of the transcription and analysis of group meetings. (3) The explicit definition of the roles of the researcher and the participants. (4) The detailed description of this study regarding the context, the participants and the development of the research. In addition, this project has followed a process of reflexivity by the researchers [75–77].

## Discussion

The definition, implementation and evaluation of strategies in caring for the family of a critical patient, during the development of the research, will result in a general, real and immediate implementation in the hospital’s ICU. Likewise, these strategies would be extrapolatable to all contexts similar to that of this study.

With regard to the applicability of the results in different contexts and fields, it is worth mentioning that, despite the limitations of the methodological design of this work, the consistency and coherence of studies carried out in dissimilar areas and contexts do lead to the perception that its application could be much broader. More specifically, if the same family needs and expectations, problems that hinder their care, and the same corresponding procedures have been detected at a national and international level, the results of this study could contribute to the development of nursing care for the families of critical patients, following prior revision and small modifications, and could be applied in other contexts.

Aside from this, the use of PAR could contribute to the incorporation of a new way of providing nursing care through reflection-action, favouring the (co)construction of nursing knowledge based on daily practices of those who would be implementing it [10, 18].

Finally, the identification of patterns of knowing and their evolution into a PAR process would increase the body of epistemological disciplinary knowledge following the feedback process between practice and theory in a professional discipline such as nursing.

### Limitations

The limitations provided for this study are as follows:

The possibility of recruiting less participants than the estimated number may result in modifications to the original design of this project. If this occurs, the criteria for forming groups would be modified, all the while ensuring the methodological adequacy of the final design.

The changes in practical nursing achieved by the participants of the study cannot be experienced by all the nurses in the unit. To maximise the distribution of strategies in clinical practice, informative briefings of the study’s results are planned out by the research team in collaboration with the participants. The research team also has the express support of the management team of critic care units to carry out this research and implement the resulting changes.

The subjective and, occasionally, unconscious nature of some patterns of knowing (e.g. aesthetic and personal) may hinder the ability for participants to identify and reflect upon them. To minimise this limitation, the researchers will produce a document, based on the bibliography, with definitions, practical examples and questions to encourage critical thinking and reflection of each of the patterns.

## Conclusion

The results of this study will allow for the implementation of substantial improvements in nursing care for families of critical patients, helping them to meet their needs and restore the family balance as well as possible, simultaneously affecting the recovery of the patient in a critical situation.

Aside from this, the use of PAR could favour the cultural change in the ICU, facilitating the effective implementation of interventions arising from the evidence obtained from daily practices. Furthermore, the study of the evolution of patterns of knowing, in the context of a participative and reflexive methodology, will provide elements of decision, both in management and in training, when selecting strategies to improve the quality of nursing practice.

## Abbreviations

ICU: Intensive Care Units
PAR: Participatory Action Research
